# RARRES2’s impact on lipid metabolism in triple-negative breast cancer: a pathway to brain metastasis

**DOI:** 10.1186/s40779-023-00480-w

**Published:** 2023-09-12

**Authors:** Quazi T. H. Shubhra

**Affiliations:** 1 Institute of Chemistry, University of Silesia in Katowice, 41-500 Chorzów, Poland; 2 Department of Neurosurgery, Renmin Hospital of Wuhan University, Wuhan, 430060 China

**Keywords:** Retinoic acid receptor responder 2 (RARRES2), Lipid metabolism, Cancer, PTEN-mTOR-SREBP1 signaling, Metabolic reprogramming, Brain metastasis

Breast cancer brain metastasis (BCBrM) is a crucial and hard area of research which guarantees an urgent need to understand the underlying molecular mechanisms. A recent study by Li et al. [1] published in *Military Medical Research* investigated the role of retinoic acid receptor responder 2 (RARRES2) in regulating lipid metabolism in BCBrM, highlighting the clinical relevance of alterations in lipid metabolites, such as phosphatidylcholine (PC) and triacylglycerols (TAGs), by RARRES2 through the modulation of phosphatase and tensin homologue (PTEN)-mammalian target of rapamycin (mTOR)-sterol regulatory element-binding protein 1 (SREBP1) signaling pathway. This commentary aims to elaborate on the key findings and their relevance to the field.

As the leading cause of death worldwide, cancer shows varied pathological progression and demands high treatment costs, posing a significant challenge to global health [2]. Breast cancer (BC), which accounted for the highest number of new diagnoses in 2020 (2.26 million new cases), exemplifies the complexity of the fight again cancers. Among the subtypes of BC, triple-negative breast cancer (TNBC) is particularly aggressive, frequently metastasizing to the brain [3] and defying most existing treatment options. This makes the management of TNBC a formidable task, marked by its propensity for rapid metastasis and limited therapeutic avenues. In this landscape, the study by Li et al. [1] gains profound significance due to offering invaluable insights into the mechanisms that drive this cancer’s spread to the brain. The brain is unique because it’s made up of a lot of fats. The study dug deeper into how BC cells that spread to the brain adapt to this fatty environment. They found that a specific protein called RARRES2 helps these cancer cells fit into the brain by changing the way they handle fats. The research illuminates the potential role of RARRES2, not only as a predictive biomarker for metastasis but also as a key to unlocking novel therapeutic strategies for this relentless form of cancer.

In addition, Li et al. [1] investigated the significant role of RARRES2 in regulating lipid metabolism within MDA-MB-231 cells. This regulation appears to directly influence BCBrM. RARRES2 has been found to interfere with the biological processes of several key metabolites, particularly PC and TAGs. Interestingly, the knockdown of RARRES2 led to an increase in PC levels and a decrease in TAGs. This discovery is not merely of academic interest but reflects on the broader mechanisms underlying dysregulated brain’s lipid metabolism. The understanding of these mechanisms is essential to unraveling the complex interactions and dependencies that characterize BC progression. Previously it was known by scientists that RARRES2 influences obesity [4] and autoimmune diseases, but this study showed that it plays a part in BC spreading to the brain as well.

The study further illuminated the role of the PTEN-mTOR-SREBP1 signaling pathway, which emerged as an important player in the regulatory network of lipid metabolism. The significance of the phosphatidylinositol 3-kinase (PI3K)/mTOR axis and the downstream SREBP signaling were highlighted as key regulatory hubs in lipid metabolism. The relationship between RARRES2 and these signaling pathways was found to be inversely proportional. RARRES2 knockdown or RARRES2 overexpression varied the protein levels of p-Akt, mTOR, p-mTOR, and SREBP1/cleaved SREBP1, establishing RARRES2’s negative regulatory effect on the mTOR-SREBP1 pathway. This negative regulation plays a critical role in controlling the complex interplay of molecules within the cancerous cells of the MDA-MB-231 line.

Another layer of complexity in the study’s findings is the role of chemokine-like receptor-1 (CMKLR1), a primary receptor through which RARRES2 exerts its biological functions. CMKLR1 knockdown could alter PTEN expression and p-Akt levels, emphasizing its relevance in the RARRES2-mediated lipid metabolic reprogramming. This revelation further clarifies the multifaceted and intricate regulatory mechanisms at play within the BC cell’s lipid metabolism. Additionally, the established positive correlation between CMKLR1 and RARRES2 aligns with and builds upon previous work, thereby integrating this study seamlessly into the broader scientific discourse [5].

The therapeutic potential of these findings was demonstrated by using the drug rapamycin (an mTOR inhibitor). When RARRES2 knockdown cells were treated with rapamycin, the increased proliferation and invasion observed in these cells were significantly reversed. This result not only corroborates the pivotal role of RARRES2 in controlling the PTEN-mTOR-SREBP1 axis but also hints at the possibility of targeted interventions that could hinder BC growth and invasion.

BC remains a common malignancy, and its effective management is vital for the health and well-being of all communities, including military personnel. Understanding the role of RARRES2 and the associated signaling pathways could inform targeted treatment strategies. The study’s findings regarding the role of RARRES2 in regulating lipid metabolism in MDA-MB-231 cells, and its interaction with the PTEN-mTOR-SREBP1 signaling pathway, provide substantial insights into the underlying mechanisms of BC. These insights have potential therapeutic implications, opening doors for targeted treatments that may ultimately benefit global BC patients. By delving into the complexities of lipid metabolic reprogramming and the interactions between various signaling pathways, the research contributes valuable knowledge to the broader understanding of BC. These findings could lead to enhanced medical strategies, not only within oncology but also in the interdisciplinary collaboration between medical fields, with potential applications in military medicine.

## Data Availability

Not applicable.

## Abbreviations

BC: Breast cancer
BCBrM: Breast cancer brain metastasis
CMKLR1: Chemokine-like receptor-1
mTOR: Mammalian target of rapamycin
PC: Phosphatidylcholine
PTEN: Phosphatase and tensin homologue
PI3K: Phosphatidylinositol 3-kinase
RARRES2: Retinoic acid receptor responder 2
SREBP: Sterol regulatory element-binding protein
TAGs: Triacylglycerols
TNBC: Triple-negative breast cancer
